# Telehealth as a Strategy to Expand Access in Brazil’s Unified Health System: Analysis of São Paulo State’s Experience Across the Three Levels of Health Care

**DOI:** 10.2196/86254

**Published:** 2026-03-20

**Authors:** Marcele S S Buto, Gabrielli B Carvalho, Michelle L Garcia, Maria Cristina C L B de Andrade, Roberta R de Lima, Giovanni G Cerri, Carlos R R Carvalho

**Affiliations:** 1 Programa de Pesquisa, Desenvolvimento e Inovação (PDI) em Saúde Digital, Hospital das Clínicas da Faculdade de Medicina da Universidade de São Paulo, São Paulo, Brazil; 2 Coordenacao de Saúde Digital, Secretaria da Saúde, São Paulo, Brazil; 3 Nucleo de Inovação Tecnologica (InovaHC), Saúde Digital, Hospital das Clínicas da Faculdade de Medicina da Universidade de São Paulo, São Paulo, Brazil; 4 Instituto de Radiologia (InRad), Hospital das Clínicas da Faculdade de Medicina da Universidade de São Paulo, São Paulo, Brazil; 5 Divisao de Pneumologia, Instituto do Coracao (InCor), Hospital das Clínicas da Faculdade de Medicina da Universidade de São Paulo, Av Dr Eneas Carvalho de Aguiar, 155, São Paulo, 05403000, Brazil, 55 1126614505

**Keywords:** telehealth, public health service, primary health care, secondary health care, tertiary health care

## Abstract

**Background:**

The COVID-19 pandemic accelerated the adoption of telehealth as a key strategy within Brazil’s Unified Health System. In São Paulo State, digital health initiatives have been developed to implement telehealth-based care models across all 3 levels of health care.

**Objective:**

The aim of this study is to describe the implementation process of the telehealth model in public health facilities to a lesser extent.

**Methods:**

This descriptive study reports on the implementation of telehealth services at the primary, secondary, and tertiary levels of health care in São Paulo State. Thirty primary health units, 4 specialty care outpatient clinics, and 18 hospitals were selected by the institutions participating in the project based on technical, health care, and infrastructure criteria. Teleconsultations were conducted through the institutional teleconferencing platform, ensuring data security and privacy. Data were collected through REDCap (Research Electronic Data Capture) between April and December 2024, including operational metrics and satisfaction scores (Net Promoter Score [NPS]). All participating health care facilities signed a term of adherence and data sharing agreement. Patients received care only after being informed and after signing a consent and adherence term for telehealth form.

**Results:**

Telehealth was implemented in 52 health care facilities across 47 municipalities in São Paulo State. A total of 19,053 teleconsultations were conducted in primary health units (NPS 97) and 218 in specialty care outpatient clinics (NPS 74), and 4178 intensive care unit case discussions were held (NPS 86).

**Conclusions:**

The findings suggest that telehealth is a feasible strategy across all levels of health care, even when implemented at a limited scale, contributing to expanded access and service coverage.

## Introduction

The structure and preparedness of international health systems experienced profound changes following the World Health Organization’s (WHO) declaration of a Public Health Emergency of International Concern in January 2020 due to the global COVID-19 pandemic [1]. This global emergency status demanded urgent action and the rapid reorganization of services, particularly in the health care sector. In this context, telemedicine emerged as a crucial tool, broadly defined as the use of Digital Information and Communication Technologies (DICT) to deliver medical services without in-person interaction between health care professionals and patients [2]. In Brazil, this scenario has intensified in recent years. In 2022, federal legislation formally regulated telehealth [3-5], highlighting its strategic role in strengthening the health care delivery network.

Beyond the immediate impacts of the pandemic, global population growth and increased life expectancy have intensified the demand for accessible, digitally integrated health care services [6]. Many of these demands have been addressed through telemedicine, particularly in the private health care sector, where its adoption has been more prevalent [7-9]. In this broader context, digital transformation and the optimization of basic health services have been recognized as viable strategies. Countries that have adopted effective public policies and strategies for digital health integration have achieved meaningful advances in health care delivery [10,11]. However, in Brazil’s public health care system, telehealth implementation still faces several barriers, including technological inequalities, economic limitations, and a shortage of trained professionals. Considering that approximately 70% of the population lacks access to private health care [12], telehealth remains relatively uncommon and not widely accessible to the entire population.

São Paulo is the most populous state in Brazil, with over 44 million inhabitants, compared to countries such as Argentina or Spain. It also holds significant economic strength, with a gross domestic product exceeding US$ 500 billion, on par with the economies of France and the United Kingdom [13,14]. This demographic and economic context positions São Paulo State as a pioneer in several fields [15-19]. Nevertheless, the state continues to face major challenges in ensuring equitable and comprehensive access to public health care. Currently, over 60% of the population, around 28 million residents, depend on the Unified Health System (Sistema Único de Saúde [SUS]) for medical services [20].

Given this scenario, particularly in low- and middle-income countries, the challenges associated with successful telehealth implementation become evident [21,22]. In addition to technological barriers and the need for interoperability between systems, key enablers of effective digital transformation include shifts in organizational culture and the empowerment of both health care professionals and the population [11,23].

In this context, the development and adoption of strategies to expand access to public health care have become urgent. In Brazil, the SUS stands to benefit significantly from digital transformation initiatives, with telehealth representing a key priority. These efforts have the potential to strengthen the health care value chain, expand service networks, and improve system performance, benefiting providers, health care professionals, and most importantly, patients [24]. Furthermore, telehealth initiatives tailored to local health needs and territorial specificities may offer an innovative approach to expanding service reach and ensuring continuity of care across all levels of the health care system [25,26].

This study aims to describe the implementation process of a telehealth model in public health care facilities, conducted on a limited scale across the 3 levels of health care.

## Methods

### Study Design

This study describes the implementation of telehealth services across 3 health care levels in São Paulo State and presents the preliminary results of this proof-of-concept, conducted between March and December 2024.

### Ethical Considerations

All participating health care facilities signed a term of adherence and data sharing form, formally confirming their participation and the sharing of individual data with the proposing health care facility. Patients seeking telehealth services at these facilities were presented with a consent and adherence term for telehealth services form and were referred to telehealth only after signing this document. The term explicitly detailed the care provisions and outlined the rights and responsibilities of both parties, including medical credentials and qualifications. This study was approved by the Research Ethics Committee for Human Subjects of Hospital das Clínicas da Faculdade de Medicina da Universidade de São Paulo (HCFMUSP; Certificate of Presentation for Ethical Review [CAAE]: 85835525.0.0000.0068, #7.366.109). The study complies with the Brazilian Data Protection Law, which sets rules and guidelines for processing and confidentiality of data [27].

### Local Context and SUS

São Paulo State is one of Brazil’s 27 federative units (26 states and 1 federal district), located in the southeastern region of the country, with a land area of 248,309.3 km² and a population of 44,651,714 inhabitants [28]. The state comprises 645 municipalities, approximately 78% of which are considered small (<50,000 inhabitants), while only 9% have more than 500,000 inhabitants; these larger municipalities account for 42% of the population, reflecting high population density in these areas [29]. São Paulo State is organized into regional divisions responsible for managing sectors, such as education, infrastructure, environment, and social development. For the health sector, the organization is structured into 17 regional health departments distributed across the territory [29].

Although the SUS is organized according to the Health Care Network (Rede de Atenção à Saúde [RAS]) model, which considers different levels of care and technological capacities to minimize fragmentation of health actions and services [30,31], challenges in integration remain. Consequently, efforts have been focused on advancing interoperability among SUS information systems [32]. São Paulo State identified an opportunity by establishing a digital health strategy aimed at expanding access to health care and promoting future integration across different health care levels.

The first step was the establishment of the *Centro Líder de Inovação em Saúde Digital*, affiliated with *Secretaria de Estado da Saúde de Sao Paulo* (Sao Paulo’s Health State Secretariat, Brazil), *Fundacao Faculdade de Medicina* (financial support), *Nucleo de Inovação Tecnologica InovaHC*, and the *Hospital das Clinicas HCFMUSP, Faculdade de Medicina, Universidade de Sao Paulo, Sao Paulo, Brazil*. Through the *Programa de Pesquisa, Desenvolvimento e Inovação em Saúde Digital*, proof-of-concept proposals for telehealth-focused care solutions were developed for integration into São Paulo State’s public health care system. Qualified professionals composed the operational and medical teams responsible for implementing the telehealth model. These teams operated at the *Centro Líder de Inovação em Saúde Digital* according to their assigned health care level.

### Implementation of Telehealth Services

The current telehealth implementation proposal was developed within the SUS organizational framework, encompassing telehealth provision in primary health units (PHUs), specialty care outpatient clinics (Ambulatório Médico de Especialidades [AMEs]), and hospitals within São Paulo State’s public health care network. Given the State’s significant territorial size and population density [33], an opportunity was identified to test a proof-of-concept aimed at assessing the feasibility of expanding telehealth across the public health care network at all 3 health care levels. The telehealth model is based on a triad: health care delivery, training program, and monitoring indicators.

For implementation, all participating health care facilities were required to meet minimum infrastructure and technological standards. These included having a private space for telehealth services, stable internet connectivity, and appropriate desktop equipment (computer, camera, microphone, and speakers). These requirements were verified through equipment functionality tests and security checks related to remote access and telehealth service delivery. All sessions were conducted using the institutional teleconferencing platform developed by HCFMUSP, ensuring data security and patients’ privacy during care delivery [34,35].

The telehealth model in primary care was designed to promote innovation and enhance health services, emphasizing health promotion through telehealth implementation at PHU. Thirty PHUs were selected based on criteria including nonparticipation in the “Mais Médicos Program”, the highest number of family health teams per unit, population size distribution, and the use of the citizen’s electronic health record.

Telehealth services were offered in the following modalities: teleconsultation (“remote medical consultations mediated by DICT, with the physician and patient located in different places”), teleinterconsultation (“exchange of information and opinions between physicians, supported by DICT, with or without the patient’s presence, to assist in clinical or surgical diagnosis or treatment”), and teleconsultancy (“consulting activities mediated by DICT among physicians, managers, and other professionals, aimed at providing clarification on administrative procedures and health actions”) [5]. These services were allocated 18 hours per week for each PHU.

**Figure 1.**
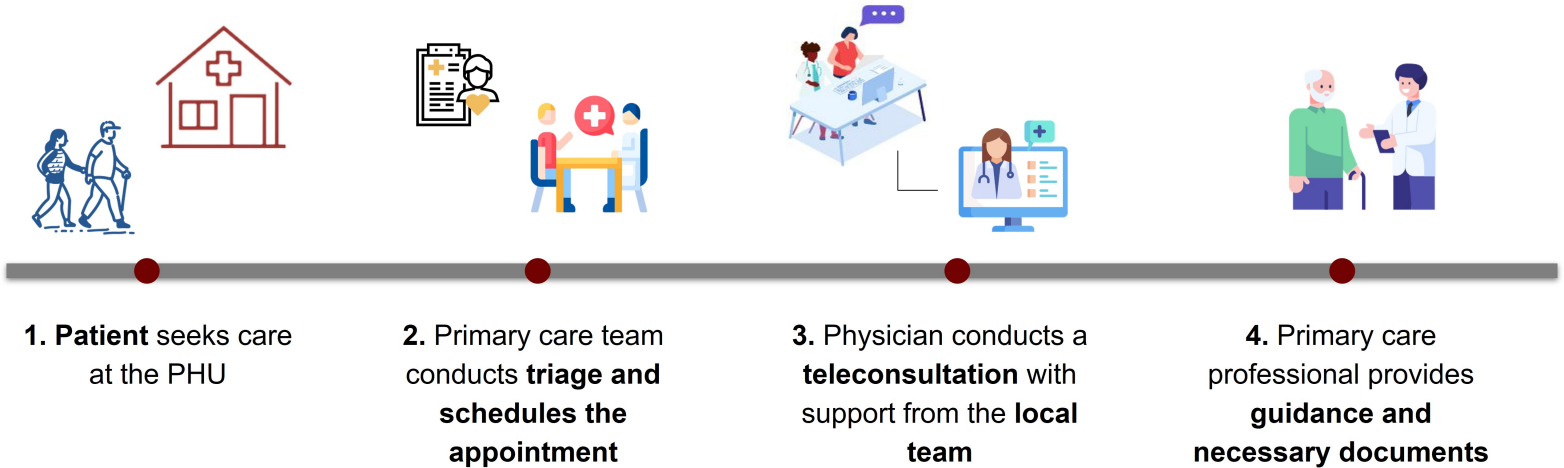
Patient’s journey at the primary health unit (PHU) within the telehealth model.

The patient’s journey began with attendance at the PHU to access the local physical and technological infrastructure, as well as to receive guidance and support from the unit’s health care professionals. The health care teams at the participating PHU were responsible for screening and scheduling patients under this model, prioritizing cases of lower clinical severity for referral. The patient’s journey is illustrated in Figure 1.

At the secondary care level, the structured model included the provision of telehealth services in 9 medical specialties: psychiatry, orthopedics, neurology, gastroenterology, cardiology, endocrinology, infectology, nephrology, and hematology. These specialties were selected based on unmet demands in the waiting lists of São Paulo State’s health care regulation system. Services were delivered through four AMEs located in different municipalities across São Paulo State. These units were selected based on having both the highest unmet demands for the selected specialties and the largest SUS-dependent population.

The telehealth strategy at this health care level involved the use of a fully remote model, designed to expand access to medical care in the 9 specialties. Teleconsultations and teleinterconsultations were offered, totaling approximately 47 hours per month per unit.

**Figure 2.**
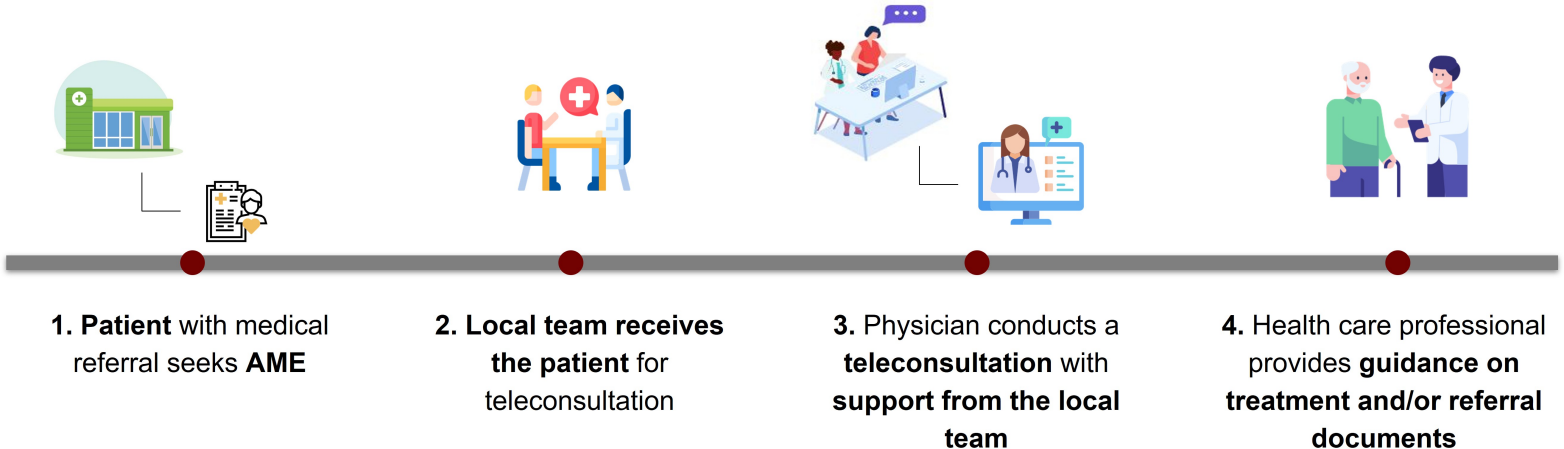
Patient’s journey at the specialty care outpatient clinic (Ambulatório Médico de Especialidades [AME]) within the telehealth model.

The patient’s journey in secondary health care began with a medical referral from primary health care. Patients were considered eligible for telehealth services based on criteria outlined in clinical protocols developed by a team of specialist physicians from HCFMUSP. These protocols aimed to guide clinical decision-making and standardize care and include elements such as *International Classification of Diseases* codes reflecting the highest demand, eligibility criteria for telehealth services, and prioritization of patients with available diagnostic test results or those who had undergone previous procedures (Figure 2).

At the tertiary health care level, the telehealth initiative aimed to provide qualified remote support to intensivist physicians and multidisciplinary teams working at intensive care unit (ICU) beds of public hospitals across São Paulo State. This support was delivered via teleinterconsultations (ICU case discussion between intensivists in a virtual environment). The exchange of knowledge in intensive care medicine and other specialties has the potential to significantly enhance hospital practices, as well as improve clinical decision-making among health care teams and administrators. Teleinterconsultations were offered in 18 selected hospitals based on criteria such as having 10 or more adult general ICU beds, having an electronic health record system implemented in the ICU, high ICU mortality rates, extended ICU length of stay, limited availability of on-staff specialists, and high clinical complexity of admitted patients. Teleinterconsultation was available 7 times per week to each participating hospital.

**Figure 3.**
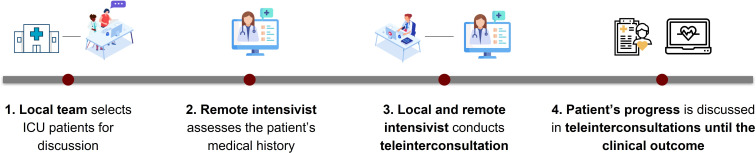
Patient’s journey in intensive care units (ICUs) within the telehealth model.

The patient’s journey at the tertiary health care level began with the selection of eligible patients for case discussion. The telemedicine intensivist first reviewed the patient’s medical history and then conducted the teleinterconsultation with the local intensivist. The patient’s clinical progress was continuously monitored and reassessed through teleinterconsultations until the final outcome of the case (Figure 3).

### Training Program

To standardize workflows and ensure consistent knowledge across teams, training programs were offered both to internal teams (physicians responsible for providing telehealth services) and to the local teams at participating health care facilities.

For the internal teams, training took place immediately after hiring and aimed to familiarize professionals with institutional clinical protocols, telehealth medical documentation, and operational tools. For the local teams, the initial training focused on familiarizing staff with telehealth workflows and platform usage. Following the start of telehealth services, the training program continued through ongoing education tailored to the specific needs of each initiative. The training program was delivered in 3 formats: asynchronous, synchronous, and in-person.

The target audience included health care professionals (physicians, nurses, nurse assistants or technicians, physical and respiratory therapists, among others) and nonclinical staff who were part of the local health care teams at participating facilities.

### Satisfaction Score

Patient and health care team satisfaction with telehealth services was evaluated using the Net Promoter Score (NPS). The NPS is derived from a single question: “On a scale from 0 to 10, how likely are you to recommend our service to a friend or colleague?” Based on the responses, the percentage of promoters (scores of 9 and 10) and detractors (scores from 0 to 6) was calculated relative to the total number of respondents. The NPS was then obtained by subtracting the percentage of detractors from that of promoters and multiplying the result by 100. A score of 76 or higher was considered to fall within the zone of excellence [36].

### Data Collection

Structured questionnaires were administered using the REDCap (Research Electronic Data Capture) platform (REDCap 14.5.21, 2025; Vanderbilt University). Data related to telehealth service delivery were collected between April 2024 and December 2024. The operational indicators are presented in Table 1.

**Table 1.** Operational metrics and indicators of the telehealth model.

Indicator	Description
Operational health care facilities	Cumulative number of units participating in the telehealth model
Number of telehealth services	Cumulative number of teleconsultations, teleinterconsultations, and teleconsultancies performed
Number of teleconsultations	Cumulative number of teleconsultations performed
Number of teleconsultancies	Cumulative number of teleconsultancies performed
Number of teleinterconsultations	Cumulative number of teleinterconsultations performed
Number of patients	Cumulative number of new patients served through telehealth
Satisfaction score (patient NPS^a^)	NPS-patient satisfaction with teleconsultation
Satisfaction score (health care team NPS)^b^	NPS-health care team satisfaction with teleinterconsultation

a NPS: Net Promoter Score.

b For hospitals, the NPS was applied to health care teams.

## Results

A total of 52 health care facilities participated in this study, covering all 3 health care levels. The characteristics of these participating health care facilities are summarized in Table 2. The participating PHU had an average population of 6366 (SD 5133.3) individuals and an average team size of approximately 13 (SD 5.1) health care professionals. Regarding the municipalities where these PHUs were located, 23 (77%) had populations of up to 50,000 inhabitants.

Among the 4 AMEs, the average team size was 90 (SD 42.1) health care professionals. In terms of the municipalities served by these AMEs, 2 (50%) were located in areas with 100,001 to 400,000 inhabitants, 1 (25%) in municipalities with 50,001 to 100,000 inhabitants, and 1 (25%) in municipalities with 10,001 to 50,000 inhabitants. Referral coverage for AME services ranged from 13 to 25 municipalities.

Regarding the 18 participating hospitals, the average team size was approximately 83 (SD 68.7) health care professionals. Based on the number of SUS beds, 15 (83.3%) were classified as large-sized hospitals (151 to 500 beds) and 3 (16.7%) as extra-large hospitals (>500 beds). In terms of administrative management, 17 (94.4%) were state-managed, while 1 (5.6%) was managed by Social Health Organizations. Among the 15 municipalities where the hospitals were located, 8 (53.3%) had populations between 100,001 and 400,000 inhabitants, 6 (40%) had more than 400,000 inhabitants, and 1 (6.7%) had between 10,000 and 50,000 inhabitants.

**Table 2.** Characteristics of the participating health care facilities (N=52).

Characteristics	Values
PHU^a^ (n=30)	
Population coverage of the PHU (mean, SD)^b^	6366.2 (5133.3)
Registered population at the PHU (mean, SD)^b^	5852.5 (3505.4)
Number of health care teams (mean, SD)^b^	1.8 (1.1)
Number of health care professionals (mean, SD)^b^	13.5 (5.1)
Municipality by population size, n (%)	
Up to 10,000 inhabitants	6 (20)
From 10,001 to 50,000 inhabitants	17 (56.7)
From 50,001 to 100,000 inhabitants	6 (20)
From 100,001 to 400,000 inhabitants	1 (3.3)
Over 400,000 inhabitants	0 (0)
Proportion of SUS^c^-dependent population per municipality, n (%)	
* *≤25%	0 (0)
25.1%‐50%	0 (0)
50.1%‐75%	8 (26.7)
75.1%‐100%	22 (73.3)
Specialty care outpatient clinics (AME) (n=4)	
Number of health care professionals (mean, SD)^b^	90.0 (42.1)
Municipality by population size, n (%)	
Up to 10,000 inhabitants	0 (0)
From 10,001 to 50,000 inhabitants	1 (25)
From 50,001 to 100,000 inhabitants	1 (25)
From 100,001 to 400,000 inhabitants	2 (50)
Over 400,000 inhabitants	0 (0)
Number of referred municipalities, n	
Botucatu	25
Dracena	17
Itapeva	15
Ourinhos	13
Hospital (n=18)	
Number of health care professionals (mean, SD)^b^^,^^d^	83.1 (68.7)
Total number of SUS beds, n (%)	
Small size (≤50 beds)	0 (0)
Medium size (51-150 beds)	0 (0)
Large size (151-500 beds)	15 (83.3)
Extra capacity (>500 beds)	3 (16.7)
Type of management, n (%)	
Municipal	0 (0)
State	17 (94.4)
Social health organization	1 (5.6)
Municipality by population size^e^, n (%)	
Up to 10,000 inhabitants	0 (0)
From 10,001 to 50,000 inhabitants	1 (6.7)
From 50,001 to 100,000 inhabitants	0 (0)
From 100,001 to 400,000 inhabitants	8 (53.3)
Over 400,000 inhabitants	6 (40.0)

a PHU: primary health unit.

b Self-reported data by the participating facility.

c SUS: Sistema Único de Saúde (Unified Health System).

d Data available for 13 hospitals.

e Includes municipalities where the participating public health care facilities were located. The city of São Paulo included 4 hospitals in the sample.

**Figure 4.**
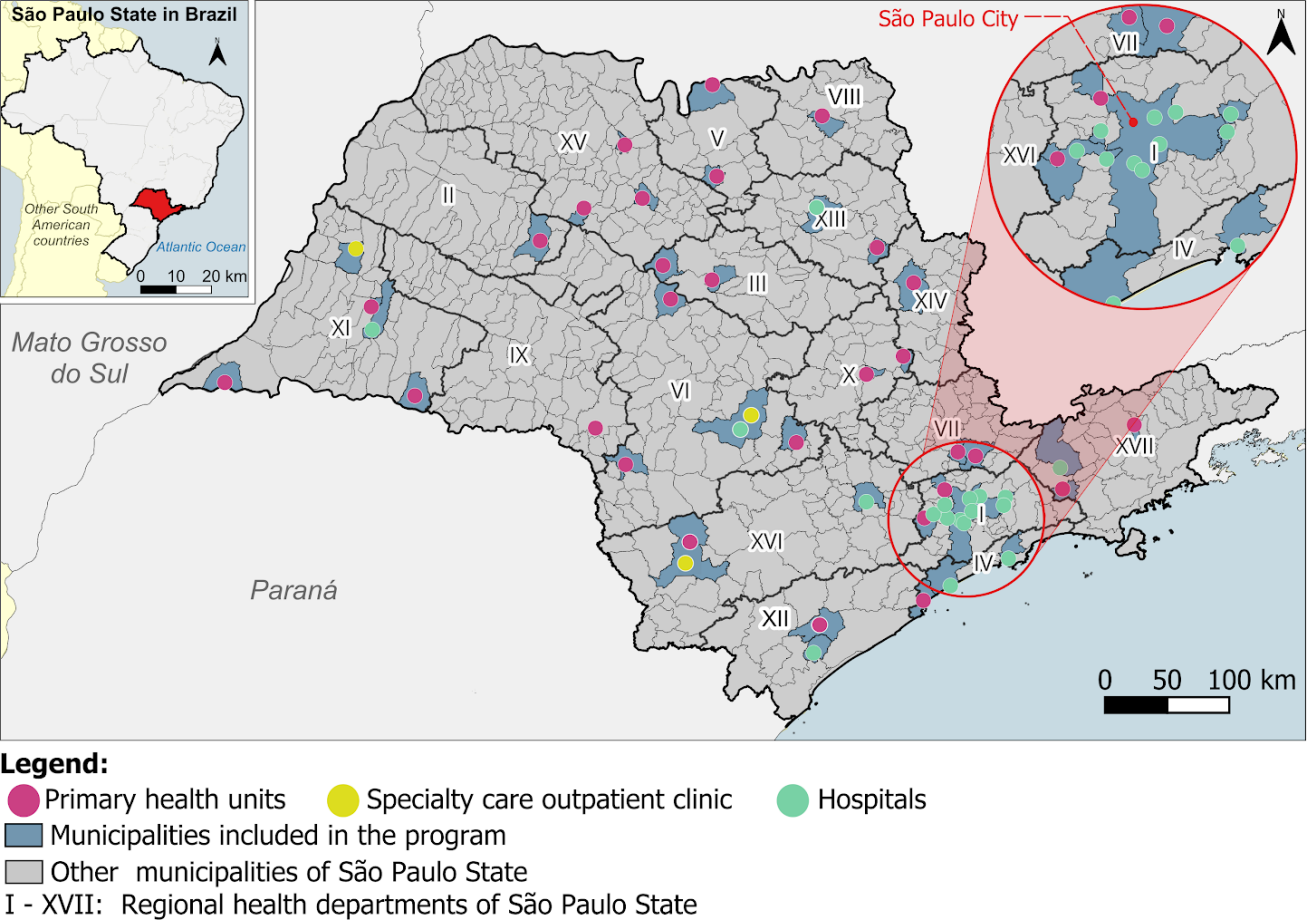
Geographic distribution of participating health care facilities by regional health departments in São Paulo State.

Regarding geographic distribution, 47 municipalities in São Paulo State were covered by at least 1 participating health care facility. The PHUs were located in 30 distinct municipalities, the AME in 4, and hospitals in 15 municipalities (Figure 4).

The main results of the operational indicators related to telehealth service provision are presented in Table 3. At the PHU level, a total of 19,041 telehealth services were delivered, comprising 14,686 teleconsultations, 3791 teleconsultancies, and 562 teleinterconsultations. A total of 12,584 patients were served, and the patient satisfaction score (NPS) was 97.0, classified within the “excellence zone”.

At the AME level, 218 telehealth services were provided, including 216 teleconsultations and 2 teleinterconsultations. A total of 218 patients were attended, with an NPS of 74, classified as the “quality zone.”

In hospitals, 4178 ICU case discussions were conducted, involving 725 patients. The health care team’s satisfaction score with the telehealth services was 86, classified within the “excellence zone.”

**Table 3.** Operational metrics of the telehealth models across primary, secondary, and tertiary health care levels in São Paulo^a^.

	PHU^b^	AME^c^	Hospital
Operation start	April 2024	November 2024	August 2024
Number of health care facilities	30	4	18
Number of telehealth services	18,874	219	—^d^
Number of teleconsultations	14,563	217	—
Number of teleconsultancies	3753	—	—
Number of teleinterconsultations	558	2	1206
Number of ICU^e^ case discussions	—	—	4654
Number of patients	12,425	219	701
Satisfaction score (NPS)^f^	97.0^g^^,^^h^	74.0	86.0^i^^,^^j^

a Data refer to the period from April 1 to December 31, 2024.

b PHU: primary health unit.

c AME: Ambulatório Médico de Especialidades (specialty care outpatient clinic).

d Not available.

e ICU: intensive care unit.

f NPS: *Net Promoter Score.*

g Patient.

h Delight zone.

i Health care team.

j Excellence zone.

## Discussion

The fragmentation of health systems and services stands out as one of the major contemporary challenges faced by public health systems worldwide [37]. In response to this issue, in 2016, the WHO proposed a significant restructuring of public health systems to its member countries [38]. This new framework emphasizes patient-centered models that integrate care across different health care levels and sectors. An opportunity was identified to encourage models aimed at the integration of health care services in order to improve service accessibility, reduce unnecessary hospitalizations and readmissions, enhance treatment adherence, increase patient satisfaction, and improve health outcomes [38].

The National Health Service in the United Kingdom is one of the most well-established public health systems globally [39]. Since its inception in 1948, the National Health Service has undergone a major legislative reform to promote a new integrated care model known as integrated care systems (ICSs) [40]. Established in 2022 across England, ICSs focus on improving health and care services through prevention, better outcomes, and the reduction of health inequalities [41]. ICSs are described as “partnerships of organizations that come together to plan and deliver joined-up health and care services and to improve the lives of people who live and work in their area.” This integration ensures holistic patient support, illness prevention, inequality reduction, and more effective management of health across primary, community, and secondary care [41].

Integration across health care levels and between health and social care sectors has also been a continuous priority in health system reforms within the Nordic countries. Each country has adopted distinct strategies to tackle challenges related to population aging, multimorbidity, and care fragmentation [42,43]. These systems are rooted in principles of universality, equity, and predominantly public financing. They are characterized by intersectoral coordination, the use of interoperable digital technologies, and multilevel governance mechanisms that foster continuity of care [42,44].

Building on these evolving health care models, telehealth has emerged as a key enabler in supporting and transforming care delivery [11]. Digital technologies are increasingly leveraged to enhance health care provision across levels of care [45]. Recent evidence has demonstrated significant benefits, including reduced hospital admissions, improved clinical outcomes, and enhanced patient experiences [46-49].

This study described the implementation process of telehealth services across all 3 health care levels in 52 public health care facilities in São Paulo State. A total of 20,464 telehealth services were delivered, covering 13,527 patients. It is important to emphasize that this initiative was designed with a limited-scale scope, aiming to implement and observe each model independently. This approach enabled a more detailed understanding of the unique characteristics and operational needs of each level of care. The insights obtained from this proof-of-concept phase are expected to inform the design of an integrated telehealth model capable of supporting patients throughout their health care journey.

In Brazil, successful telemedicine experiences have been reported across different population groups and health conditions within the SUS at all 3 health care levels [50-53]. In 2021, the Digital Primary Health Care (APS Digital) initiative was implemented in the remote community of Paissandú district (Santarém, Pará State, Brazil). In this initiative, a family and community physician based at HCFMUSP conducted teleconsultations, supported by a local health team consisting of a nurse and 2 nursing technicians. Over a 3-month period, the initiative delivered 220 teleconsultations, achieving 95% patient satisfaction and resolving 76.6% of medical complaints, highlighting the viability of telehealth in underserved areas [50].

Also at the primary care level, a similar initiative led by HCFMUSP, in partnership with the Brazilian Agency for Support to the Management of the Unified Health System, launched the UBS+Digital project. The aim was to provide teleconsultations in PHUs lacking on-site physicians, with 18 weekly hours allocated to telehealth services. Over a 9-month period, 6312 teleconsultations were conducted, and the patient satisfaction score (NPS) reached 97, placing the initiative within the excellence zone [53].

It is worth highlighting that, in these earlier initiatives, PHUs did not have physicians as part of their health care teams. In contrast, this study specifically selected PHUs with on-site physicians, which enabled the implementation of additional telehealth modalities, such as teleinterconsultations. These sessions facilitate the exchange of clinical information and opinions between physicians to support diagnostic and therapeutic decisions [54]. In addition, the presence of a full health care team allowed the use of teleconsultancy, which supports administrative and clinical decision-making through interactions among professionals. Together, these two modalities accounted for approximately 23% of all telehealth services delivered in PHUs, suggesting that collaborative knowledge-sharing is becoming an increasingly embedded practice in primary care, one that enhances service management, team performance, and clinical outcomes [54,55].

In this study, the municipalities where participating PHUs were located had more than 50% of their populations depending on SUS. This scenario is consistent with São Paulo State’s overall primary care coverage rate of 68.4%, indicating that many regions still face challenges in accessing timely and comprehensive primary health care services [56]. In this context, telehealth emerges as a crucial strategy for expanding access and reducing inequities at the primary care level.

Regarding secondary health care, AMEs were established in São Paulo State in 2007 as high-resolution health care facilities equipped with modern technologies, offering consultations, diagnostic tests, and in some cases, surgical procedures all within a single location [57]. Patients referred to AME come from primary health care services to address more complex health needs that could not be fully managed at the primary level. For the current telehealth model, 4 AMEs were selected, each offering an average of 47 hours of telehealth services per month. A total of 218 telehealth services were delivered, with a satisfaction score (NPS) of 74, which falls within the “quality zone.”

Studies describing telehealth initiatives through AME remain scarce in the literature. In contrast, primary health care has increasingly been recognized as a favorable setting for integrating specialized care via telehealth projects. The “TeleAMES” project, approved by the Brazilian Ministry of Health, facilitated over 142,000 teleinterconsultations across 12 specialties at PHUs and Indigenous Health Centers in the North and Central-West regions of Brazil [58]. Similarly, other initiatives integrating specialist physicians into primary health care settings through teleinterconsultations and teleconsultancies have reported positive outcomes in terms of referral quality, patient satisfaction, and continuity of care [59-61].

Within this context of specialized health care delivery, even though implemented to a lesser extent, the telehealth model adopted in the AME of São Paulo State enabled scheduling of medical appointments across 9 specialties, reflecting the highest demand identified among the general population. The volume of telehealth services provided was dependent on scheduling availability within the Health Care Regulation System of São Paulo State, a platform designed to manage the allocation of specialized health care appointments and optimize population access to secondary health care services [62].

Initiatives in secondary health care aim to address persistent challenges in public health systems, such as inefficiencies in regulatory mechanisms, prolonged waiting lists, and the uneven distribution of medical specialists across geographic areas [58,63]. Access to secondary care services is consistently identified as one of the primary barriers to achieving comprehensive care within the SUS and is often described as the main “bottleneck” in the implementation of the RAS model [63-65]. In this context, initiatives that support the structuring and planning of strategies to alleviate pressures on the public health system and promote best practices are essential for the ongoing development of the SUS and the enhancement of user experience.

At the tertiary care level in the present study, a total of 4178 ICU case discussions were conducted through 1205 teleinterconsultations, serving 725 patients across 18 public hospitals in São Paulo State. The health care team satisfaction score was 86, placing it within the “excellence zone.” Several hospital-based telehealth initiatives have gained prominence, especially during the COVID-19 pandemic. The HCFMUSP led the implementation of hospital telehealth services and the TeleICU model, which included online training for health care professionals. The Tele-ICU-SES project completed 11,823 teleinterconsultations across 34 hospitals in São Paulo, achieving a satisfaction score of 82. Its subsequent expansion to 5 additional hospitals nationwide (Tele-ICU-Brazil) resulted in 936 ICU case discussions and over 11,500 teleinterconsultations. Due to the increased risk among pregnant patients, particularly vulnerable to severe COVID-19, the project was further extended as Tele-ICU-Obstetric, providing telehealth support in 27 hospitals throughout the country [52]. At the hospital level, other methodologies were developed and adapted to broaden telehealth services across multiple specialties, totaling 140,671 telehealth services and meeting expectations for teleconsultation quality [51].

Telehealth services in ICUs are recognized as a form of organizational innovation that enhances access to and quality of critical care [66]. Importantly, the effectiveness of these interventions is closely linked to the characteristics of the local environment and the degree of collaboration between remote providers and on-site critical care teams [66,67]. Therefore, understanding the local health care culture, available resources, and stimulating engagement among local health care professionals is crucial. In this study, the satisfaction level of participating health care teams reached the “excellence zone,” reflecting high acceptance of the telehealth model implemented.

Finally, it is important to acknowledge the heterogeneity of public health care facilities in Brazil, particularly regarding resource availability across different regions and levels of care [68,69]. In this context, the successful implementation of innovative care models, such as telehealth, is often challenged by structural limitations, including insufficient infrastructure, unstable internet connectivity, and limited familiarity of health care professionals with digital tools. In this study, some participating health care facilities reported difficulties related to the availability of electronic equipment (computer, webcam, microphone, and headset), lack of private spaces suitable for remote consultations, and unstable internet connectivity. In these situations, the support provided by higher administrative levels proved essential to bridging such gaps and ensuring the successful execution of the telehealth model.

Since 2021, the WHO has been actively supporting countries in adopting digital health strategies to expand health care access [11]. These efforts align with the Sustainable Development Goals of the United Nations 2030 Agenda [70,71]. Within this framework, one of the guiding principles of digital transformation emphasizes the importance of inclusivity, aiming to reach not only socioeconomically, geographically, or culturally vulnerable populations but also those affected by digital illiteracy [72]. In this regard, DICT holds significant potential to reduce health inequalities by facilitating timely access to health information and digital tools for prevention and care [72].

Digital transformation in health care is a continuous process that requires not only the integration of new technologies but also a profound shift in organizational culture to foster a more efficient, accessible, and innovative health system. Although persistent challenges remain, including technological, cultural, and social barriers, as well as issues related to service and resource management [73], the main findings of this study are consistent with the existing literature and reinforce the understanding that telehealth represents a promising and feasible strategy to strengthen the SUS.

The findings and reflections presented in this study suggest the feasibility of implementing telehealth services across all 3 health care levels. Although applied on a limited scale, the initiative contributed to expanding access to public health care, reaching diverse regions throughout São Paulo State, and achieving favorable satisfaction scores among both patients and health care professionals.

Considering the geographic extent and regional disparities within São Paulo State, future telehealth strategies should be grounded in a comprehensive situational assessment of each territory, including evaluations of cultural context, technological infrastructure, availability of human resources, digital literacy, and the need for continuous professional training.

In this regard, the model implemented and described in this study**—**grounded in the triad of health care delivery, training program, and monitoring indicators—demonstrates significant potential for sustainable scale-up and reinforces the role of telehealth as a strategic and impactful approach to strengthening the public health care system.

## Abbreviations

AME: Ambulatório Médico de Especialidades (specialty care outpatient clinic)
DICT: Digital Information and Communication Technologies
HCFMUSP: Hospital das Clínicas da Faculdade de Medicina da Universidade de São Paulo
ICS: integrated care systems
NPS: Net Promoter Score
PHU: primary health unit
RAS: Rede de Atenção à Saúde (Health Care Network)
REDCap: Research Electronic Data Capture
SUS: Sistema Único de Saúde (Unified Health System)
WHO: World Health Organization

## References

[1] WHO director-general’s opening remarks at the media briefing on COVID-19 - 11 March 2020. World Health Organization.

[2] Messias JR, Pacheco GG, Faria BO (2023). Telemedicina durante a pandemia do COVID-19 [Article in Portuguese]. Braz J Implantol Health Sci.

[3] (2022). Resolução CFM n° 2.314/2022, de 5 de maio de 2022. define e regulamenta a telemedicina, como forma de serviços médicos mediados por tecnologias de comunicação [Report in Portuguese]. https://abmes.org.br/arquivos/legislacoes/Resolucao-CFM-2314-2021-04-20.pdf.

[4] (2022). Portaria GM/MS n° 1348, de 2 de junho de 2022 dispõe sobre as ações e serviços de telessaúde no âmbito do sistema único de saúde (SUS) [Report in Portuguese]. https://www.foa.unesp.br/Home/pos/ppgops/portaria-gm-ms-n-1.348-de-2-de-junho-de-2022.pdf.

[5] (2022). Lei nº 14.510, de 27 de dezembro de 2022 – Altera a Lei nº 8.080/1990 e Lei nº 13.146/2015, e revoga a Lei nº 13.989/2020 [Web page in Portuguese]. Presidência da República.

[6] (2023). The role of digital technologies in aging and health. https://www.itu.int/cities/wp-content/uploads/2023/04/The-role-of-Digital-Technologies-in-Aging-and-Health.pdf.

[7] Scheffer M, Cassenote A, de Britto E Alves MTSS, Russo G (2022). The multiple uses of telemedicine during the pandemic: the evidence from a cross-sectional survey of medical doctors in Brazil. Global Health.

[8] Amaral JLG, Endrigo AC, Malik AM, Ogata AJN, Assumpção Neto JC (2022). Reactions of physicians in the state of São Paulo to the use of telemedicine during the SARS-CoV-2 pandemic: cross-sectional study. Sao Paulo Med J.

[9] Barbosa PM, Silva Júnior F da, Lima GMC (2022). Using data from a private provider of telemedicine to assess the severity of the early 2021 COVID-19 wave in Brazil. Braz J Med Biol Res.

[10] Hecht J (2019). The future of electronic health records. Nature New Biol.

[11] (2021). Global strategy on digital health 2020-2025. https://www.who.int/docs/default-source/documents/gs4dhdaa2a9f352b0445bafbc79ca799dce4d.pdf.

[12] (2020). Pesquisa nacional de saúde 2019: informações sobre domicílios, acesso e utilização dos serviços de saúde [Report in Portuguese]. https://biblioteca.ibge.gov.br/visualizacao/livros/liv101748.pdf.

[13] (2022). Produto interno bruto (PIB) – anual [Report in Portuguese]. https://pib.seade.gov.br/anual.

[14] GDP (current US$). World Bank Group.

[15] (2025). Investimentos em São Paulo estimulam mobilidade sustentável [Report in Portuguese]. https://informa.seade.gov.br/wp-content/uploads/2025/10/seade-informa-economia-investimentos-sao-paulo-estimulam-mobilidade-sustentavel.pdf.

[16] (2025). Evolução do número de empresas entre 2014 e 2024 [Report in Portuguese]. https://informa.seade.gov.br/wp-content/uploads/2025/04/seade-informa-economia-evolucao-numero-empresas-2014-2024.pdf.

[17] (2025). Investimentos ampliam o complexo da saúde de SP no pós‑pandemia [Report in Portuguese]. https://informa.seade.gov.br/wp-content/uploads/2025/05/seade-informa-economia-investimentos-ampliam-complexo-saude-sao-paulo.pdf.

[18] (2025). Evolução da urbanização da população paulista [Report in Portuguese]. https://informa.seade.gov.br/wp-content/uploads/2025/02/seade-informa-demografia-urbanizacao-populacao-populacao-paulista.pdf.

[19] (2024). Perfil dos municípios paulistas em 2023 [Report in Portuguese]. https://informa.seade.gov.br/wp-content/uploads/2024/09/seade-informa-demografia-perfil-municipios-paulistas-2023.pdf.

[20] (2024). Estimativa da população SUS dependente (com base na saúde suplementar) atualizado em: 30/08/2025. Sao Paulo’s Health State Secretariat, Brazil.

[21] Ye J, He L, Beestrum M (2023). Implications for implementation and adoption of telehealth in developing countries: a systematic review of China’s practices and experiences. NPJ Digit Med.

[22] Scott Kruse C, Karem P, Shifflett K, Vegi L, Ravi K, Brooks M (2018). Evaluating barriers to adopting telemedicine worldwide: a systematic review. J Telemed Telecare.

[23] Gonçalves RL, Pagano AS, Reis ZSN (2023). Usability of telehealth systems for noncommunicable diseases in primary care from the COVID-19 pandemic onward: systematic review. J Med Internet Res.

[24] (2020). Estratégia de saúde digital para o brasil 2020‑2028 [recurso eletrônico] [Report in Portuguese]. https://bvsms.saude.gov.br/bvs/publicacoes/estrategia_saude_digital_Brasil.pdf.

[25] Catapan SC, Melo EA, Silva AB, Albuquerque MV, Calvo MCM (2024). Teleassistência no Sistema Único de Saúde brasileiro: onde estamos e para onde vamos [Article in Portuguese]. Ciênc Saúde Colet.

[26] Cezário LRA, Ferreira BF, Manoel AV (2024). Telessaúde no Brasil: uma revisão de escopo [Article in Portuguese]. Rev Baiana de Saúde Pública.

[27] (2019). LEI Nº 13.853, DE 8 DE JULHO DE 2019: Altera a Lei nº 13.709, de 14 de agosto de 2018, para dispor sobre a proteção de dados pessoais e para criar a Autoridade Nacional de Proteção de Dados; e dá outras providências [Web page in Portuguese]. Presidência da República.

[28] Seade População [Website in Portuguese].

[29] (2023). Diagnóstico preliminar do estado de são paulo: desafios para o planejamento plurianual do período de 2024-2027 [Report in Portuguese]. http://planejamento.sp.gov.br/static/arquivos/ppa/ppa2027/DIAGNOSTICO_PRELIMINAR_DO_ESTADO_DE_SAO_PAULO.pdf.

[30] (2010). PORTARIA Nº 4.279, DE 30 DE DEZEMBRO DE 2010 — Estabelece diretrizes para a organização da Rede de Atenção à Saúde no âmbito do Sistema Único de Saúde (SUS) [Web page in Portuguese]. Ministério da Saúde.

[31] (2022). Curso I: regulação de sistemas de saúde do SUS: módulo 4: redes de atenção à saúde [recurso eletrônico] [Report in Portuguese]. https://bvsms.saude.gov.br/bvs/publicacoes/aula4_regulacao_redes_atencao_saude.pdf.

[32] (2025). Decreto nº 12.560, de 23 de julho de 2025. Dispõe sobre a Rede Nacional de Dados em Saúde e sobre as Plataformas SUS Digital e regulamenta o art. 47 e o art. 47‑A, caput, § 1 [Web page in Portuguese]. Presidência da República.

[33] (2023). Cidades e estados [Web page in Portuguese]. Instituto Brasileiro de Geografia e Estatística (IBGE).

[34] Furuie SS, Rebelo MS, Moreno RA (2007). Managing medical images and clinical information: InCor’s experience. IEEE Trans Inf Technol Biomed.

[35] Macedo BR, Garcia MVF, Garcia ML (2021). Implantação de telemedicina de terapia intensiva durante a pandemia de COVID-19 [Article in Portuguese]. J Bras Pneumol.

[36] Reichheld FF (2003). The one number you need to grow. Harv Bus Rev.

[37] Pan American Health Organization (PAHO) Policy on integrated care for improved health outcomes. https://www.paho.org/sites/default/files/csp30-10-e-policy-integrated-care_0.pdf.

[38] (2016). Strengthening integrated, people-centred health service. https://apps.who.int/gb/ebwha/pdf_files/WHA69/A69_R24-en.pdf.

[39] Grosios K, Gahan PB, Burbidge J (2010). Overview of healthcare in the UK. EPMA J.

[40] Dunn P, Fraser C, Williamson S, Alderwick H (2022). Integrated care systems: what do they look like?. https://www.health.org.uk/sites/default/files/pdf/2022-07/2022%20-%20ICS%20characteristics_v3.pdf.

[41] What are integrated care systems. NHS England.

[42] European Observatory on Health Systems and Policies (2024). Denmark: health system review. https://iris.who.int/server/api/core/bitstreams/b0992867-dee5-45fa-80b6-1e008fd353c0/content.

[43] Magnussen J, Vrangbæk K, Saltman RB (2009). Nordic Health Care Systems: Recent Reforms and Current Policy Challenges.

[44] (2024). Organization and responsibility for the health sector. Nordic Health and Welfare Statistics.

[45] Allcock JA, Zhuang M, Li S, Zhao X (2024). Landscape of digital technologies used in the National Health Service in England: content analysis. JMIR Form Res.

[46] (2024). Summary of south east region virtual wards evaluation. NHS England.

[47] (2024). Remote consulting. NHS England.

[48] Shao Y, Shi L, Nauman E, Price-Haywood E, Stoecker C (2024). Telehealth use and its impact on clinical outcomes in patients with type 2 diabetes during the COVID-19 pandemic. Diabetes Obes Metab.

[49] Snoswell CL, Chelberg G, De Guzman KR (2023). The clinical effectiveness of telehealth: a systematic review of meta-analyses from 2010 to 2019. J Telemed Telecare.

[50] Bin KJ, Santana Alves PG, Costa R (2023). User experience regarding digital primary health care in Santarém, Amazon: evaluation of patient satisfaction and doctor’s feedback. JMIR Form Res.

[51] Scudeller PG, Pereira AJ, Cerri GG (2023). Telemedicine in Brazil: teleconsultations at the largest university hospital in the country. Telemed Rep.

[52] Scudeller PG, Lamas CA, Alvarenga AM (2023). Tele-intensive care unit program in Brazil: implementation and expansion. Telemed Rep.

[53] Lamas CA, Santana Alves PG, Nader de Araújo L (2025). Telehealth initiative to enhance primary care access in Brazil (UBS+Digital Project): multicenter prospective study. J Med Internet Res.

[54] Menezes LL, Souza MIC, Simas KBF (2024). Análise da percepção de médicos do Sistema Único de Saúde sobre o uso da teleinterconsulta em Campo Grande-MS, Brasil [Articlein Portuguese]. Ciênc Saúde Colet.

[55] Ahmed A, Mutahar M, Daghrery AA (2024). A systematic review of publications on perceptions and management of chronic medical conditions using telemedicine remote consultations by primary healthcare professionals April 2020 to December 2021 during the COVID‑19 pandemic. Med Sci Monit.

[56] (2024). Histórico de Cobertura - APS [Web page in Portuguese]. Ministério da Saúde e-Gestor Atenção Primária à Saúde.

[57] Ambulatório médico de especialidades (AMES) [Web page in Portuguese]. Secretaria de Estado da Saúde, Estado de São Paulo.

[58] TeleAMES assistência médica especializada nas regiões norte e centro-oeste do brasil por meio de telemedicina [Web page in Portuguese]. Hospitais PROADI‑SUS.

[59] (2011). Portaria n° 2546, de 27 de outubro de 2011 redefine e amplia o programa telessaúde brasil, que passa a ser denominado programa nacional telessaúde brasil redes (telessaúde brasil redes) [Report in Portuguese]. https://bvsms.saude.gov.br/bvs/saudelegis/gm/2011/prt2546_27_10_2011.html.

[60] Chagas MEV, Fernandes GR, Fernandes DH (2025). Assistência médica especializada na atenção primária por meio da telemedicina no Nordeste do Brasil: estudo descritivo, Rio Grande do Norte, 2022-2023 [Artilce in Portuguese]. Epidemiol Serv Saúde.

[61] Maeyama MA, Calvo MCM (2018). A Integração do Telessaúde nas Centrais de Regulação: a Teleconsultoria como Mediadora entre a Atenção Básica e a Atenção Especializada [Article in Portuguese]. Rev bras educ med.

[62] (2024). Recursos em saúde: caderno 3 – exames (capítulo 1) coordenadoria de regiões de saúde [Report in Portuguese]. https://www.saude.sp.gov.br/resources/ses/perfil/gestor/homepage//caderno_1-consultas_nas_especialidades_-_2_versao_atualizada_ago_2024.pdf.

[63] Erdmann AL, de Andrade SR, de Mello A, Frago LC (2013). A atenção secundária em saúde: melhores práticas na rede de serviços [Article in Portuguese]. Rev Latino‑Am Enferm.

[64] Oliveira RC, Correa AA, Ferreira AG, Marques ZFA, Magalhães HM (2010). Desafios e Inovações Na Gestão Do SUS Em Belo Horizonte: A Experiência de 2003 A 2008 [Book in Portuguese].

[65] Spedo SM, Pinto NR, Tanaka OY (2010). O difícil acesso a serviços de média complexidade do SUS: o caso da cidade de São Paulo, Brasil [Article in Portuguese]. Physis.

[66] Vranas KC, Slatore CG, Kerlin MP (2018). Telemedicine coverage of intensive care units: a narrative review. Ann Am Thorac Soc.

[67] Becker C, Frishman WH, Scurlock C (2016). Telemedicine and Tele-ICU: the evolution and differentiation of a new medical field. Am J Med.

[68] Paim JS (2018). Sistema Único de Saúde (SUS) aos 30 anos [Article in Portuguese]. Ciênc saúde colet.

[69] Rache B, Rocha R, Nunes L, Spinola P, Malik AM, Massuda A (2020). Necessidades de infraestrutura do SUS em preparo ao COVID19: leitos de UTI, respiradores e ocupac¸ao hospitalar [Report in Portuguese]. https://www.epsjv.fiocruz.br/sites/default/files/files/NT3%20vFinal.pdf.

[70] (2015). Transforming our world: the 2030 agenda for sustainable development. https://docs.un.org/en/A/RES/70/1.

[71] Novillo-Ortiz D, De Fátima Marin H, Saigí-Rubió F (2018). The role of digital health in supporting the achievement of the Sustainable Development Goals (SDGs). Int J Med Inform.

[72] (2021). Eight guiding principles of digital transformation of the health sector. A call to pan American action. https://iris.paho.org/server/api/core/bitstreams/1af90228-5e3f-41ab-82a9-771aba475019/content.

[73] Meskó B, Drobni Z, Bényei É, Gergely B, Győrffy Z (2017). Digital health is a cultural transformation of traditional healthcare. mHealth.

[74] ChatGPT overview. OpenAI.

